# No difference in outcomes between large- and small-pore meshes in a prospective, randomized, multicenter trial investigating open retromuscular meshplasty for incisional hernia repair

**DOI:** 10.1007/s00423-022-02751-x

**Published:** 2023-01-13

**Authors:** Andreas Kroh, Markus Zufacher, Roman Eickhoff, Daniel Heise, Marius Helmedag, Florian Ulmer, Ulf P. Neumann, Joachim Conze, Ralf-Dieter Hilgers, Marcel Binnebösel

**Affiliations:** 1 Department of General, Visceral and Transplantation Surgery, RWTH Aachen University Hospital, Aachen, Germany; 2 Department of General and Visceral Surgery, Klinikum Bielefeld, University Hospital OWL of the University of Bielefeld, Bielefeld, Germany; 3 Department of Surgery, Maastricht University Medical Center (MUMC), Maastricht, The Netherlands; 4 UM Hernienzentrum Dr. Conze, Arabella Klinikum München, Munich, Germany; 5 Institute for Medical Statistics, RWTH Aachen University Hospital, Aachen, Germany

**Keywords:** Incisional hernia, Retromuscular meshplasty, Small-pore mesh, Large-pore mesh

## Abstract

**Abstract:**

**Study design:**

A randomized, controlled, prospective multicenter clinical trial with a parallel group design was initiated in eight surgical centers to compare a large-pore polypropylene mesh (Ultrapro^®^) to a small-pore polypropylene mesh (Premilene^®^) within a standardized retromuscular meshplasty for incisional hernia repair.

**Methods:**

Between 2004 and 2006, patients with a fascial defect with a minimum diameter of 4 cm after vertical midline laparotomy were recruited for the trial. Patients underwent retromuscular meshplasty with either a large-pore or a small-pore mesh to identify the superiority of the large-pore mesh. Follow-up visits were scheduled at 5 and 21 days and 4, 12, and 24 months after surgery. A clinical examination, a modified short form 36 (SF-36^®^), a daily activity questionnaire, and an ultrasound investigation of the abdominal wall were completed at every follow-up visit. The primary outcome criterion was foreign body sensation at the 12-month visit, and the secondary endpoint criteria were the occurrence of hematoma, seroma, and chronic pain within 24 months postoperatively.

**Results:**

In 8 centers, 181 patients were included in the study. Neither foreign body sensation within the first year after surgery (27.5% Ultrapro^®^, 32.2% Premilene^®^) nor the time until the first occurrence of foreign body sensation within the first year was significantly different between the groups. Regarding the secondary endpoints, no significant differences could be observed. At the 2-year follow-up, recurrences occurred in 5 Ultrapro^®^ patients (5.5%) and 4 Premilene^®^ patients (4.4%).

**Conclusion:**

Despite considerable differences in theoretical and experimental works, we have not been able to identify differences in surgical or patient-reported outcomes between the use of large- and small-pore meshes for retromuscular incisional hernia repair.

**Trial registration:**

Clinical Trials NCT04961346 (16.06.2021) retrospectively registered.

## Introduction

Incisional hernia remains a continuous problem in surgery. Although great efforts have been undertaken to optimize the primary closure of a laparotomy, the number of incisional hernia repairs has remained steady over the past decade, with almost 50,000 repairs in Germany each year. However, although retromuscular hernia repair is considered the gold standard, different surgical techniques are used. To date, a vast number of prospective randomized trials dealing with incisional hernia repair have been published [1]. While there are an increasing number of randomized controlled trials (RCT) or guidelines investigating laparoscopic hernia repair [2–6] or prophylactic prosthetic reinforcement of midline abdominal incisions [7–9], there are still few data regarding the open sublay technique [1]. This is mostly due to the poor quality of several meta-analyses, systematic reviews, and RCTs, in which patients with incisional and primary ventral hernias are often pooled and surgical techniques are not standardized between studies. This was shown in a previously performed multicenter trial comparing large-pore meshes vs. conventional meshes for incisional hernia repair. In that study, there was an unexpectedly high incidence of recurrence, with an overall rate of 12% after 2 years of follow-up [10]. The critical analysis of these results revealed considerable impairments in the study protocol, leaving many important steps of the surgical technique to each center’s standard procedure. Consequently, the present study was initiated to abolish these shortcomings, comparing only two different meshes, i.e., a small-pore polypropylene mesh (Premilene^®^) and a large-pore composite mesh (Ultrapro^®^), within a completely standardized, retromuscular mesh augmentation. Meshes made from polypropylene have a tensile strength that is far greater than that required physiologically [11]. Reducing the amount of polypropylene by increasing the pore size produces a lighter weight mesh. Large-pore composite meshes are the result of incorporating an absorbable component into a reduced polypropylene mass [12], leading to improved functional properties that may diminish mesh-related complications [13]. However, although mesh reinforcement in hernia repair is widely accepted and the literature reports on many experimental data regarding different mesh types [14–18], there is still the need for high-quality RCTs reporting surgical and patient-related outcomes on different meshes. Thus, the aim of the study was to perform a prospective randomized multicenter study comparing a small-pore and large-pore composite mesh in a standardized open retromuscular meshplasty.

## Material and methods

### Study design

This study was conducted in accordance with the CONSORT statement [19] as a randomized, controlled, prospective multicenter clinical trial comparing two meshes (Ultrapro^®^ and Premilene^®^) within a parallel group design to identify the superiority of the large-pore composite mesh. Eight surgical centers from Germany participated in this trial. Institutional review board (IRB)/ethics committee approval was obtained from all participating centers (EK 062/04). The study is registered in the ClinicalTrials.gov register (https://clinicaltrials.gov) under NCT04961346. Randomization was achieved by computer-generated random numbers in sealed envelopes with block sizes to ensure balanced recruitment within each center. All patients gave written informed consent before participation in the trial. The inclusion criteria were an age of 18 years and older with a fascial defect after vertical midline laparotomy of a minimum of 4 cm in diameter. Patients were excluded when the hernia was incarcerated, recurrent after mesh repair, or when the hernia was not in the midline. Additionally, patients with a malignancy or chemotherapy within the last 3 months, pregnancy, participation in other studies, and wound infection were excluded from this trial. Patients were recruited between 2004 and 2006. The study was observer and patient blinded such that the study staff performed the postoperative assessments and the patients were unaware of treatment allocation.

### Baseline characteristics and clinical follow-up

Recruitment was followed by documentation of the medical history, occupation, and body mass index (BMI) according to the case report form (CRF). Before surgery, the questionnaires for the short form 36 (SF-36^®^) and daily activity were completed by the patient. All patients agreed to be examined at five scheduled follow-up visits: 5 days after surgery; 21 days after surgery; and 4, 12, and 24 months after surgery. Additional examinations were performed in the case of reoperations or at any unscheduled visit (e.g., because of concomitant therapy or adverse events). In addition to a clinical examination, a modified SF-36®, a daily activity questionnaire, and an ultrasound investigation of the abdominal wall were completed at each visit.

### Outcomes

The primary outcome criterion was foreign body sensation assessed by the patient’s judgment regarding the question “Do you feel the mesh at rest?” at the 12-month follow-up visit. To exclude foreign body sensation related to surgical trauma, foreign body sensation was defined as a persistence of a minimum of 3 months. The analysis of the outcome was based on the time until the first occurrence of foreign body sensation within the first year after surgery. It is further specified by the question of whether the foreign body sensation was distracting and interfered with quality of life. The secondary endpoint criteria were the occurrence of postoperative seroma within 24 months, hematoma at the 5-day visit, hematoma requiring surgery at the 5-day visit, and chronic pain as well as recurrence after 24 months. Hematoma and seroma dimensions were determined by b-mode ultrasound. Chronic pain was evaluated by a questionnaire containing questions related to the time point, character, localization, and trigger of pain. A quantitative assessment of chronic pain was performed using a visual analog scale (VAS, scale: 0–10). Quality of life was investigated by the SF-36^®^ scoring system. The scores of the 36 items of this questionnaire can be condensed into two different summary scale scores: the physical component summary (PCS), reflecting physical life quality, and the mental component summary (MCS), reflecting mental life quality.

### Meshes

Two different monofilament polypropylene meshes were investigated in this study. Ultrapro^®^ (Ethicon Inc., Somerville, NJ, USA) is a large-pore polypropylene mesh with additional absorbable poliglecaprone. The absorption of the poliglecaprone component takes up to 120 days. Premilene^®^ (Atrium Medical, Mijdrecht, The Netherlands) is a small-pore, polypropylene mesh. Detailed characteristics of both meshes are summarized in Table 1.

**Table 1:** Characteristics of mesh material used for incisional hernia repair in this trial

	Ultrapro^®^	Premilene^®^
Material	Polypropylene & Poliglecaprone (resorption within 120 days)	Polypropylene
Weight	28 g/m^2^	82 g/m^2^
Thickness	0.5 mm	0.5 mm
Pore size	3–4 mm	0.8 mm
Filament thickness	0.1 mm	0.15 mm
Tensile strength	35 N/cm (polypropylene)	101 N/cm

### Operative details

All steps of the operation were predefined and performed in a standardized surgical procedure. After disinfection and sterile covering of the surgical site, the former skin scar was excised over the total length. The next step was the preparation of the hernia sac and exposure of the fascia defect. The hernia sac was opened, and an incision of the total fascia scar under vision was performed. After dissolving local adhesions to the abdominal wall, the preparation of the mesh layer started with opening the rectus sheath as medially as possible. The retromuscular space was developed at least 5–6 cm to each side. At the cranial and caudal edges of the fascial defect, the posterior rectus sheath was incised on both sides close to the linea alba over a minimum length of 5 cm with preparation of the “fatty triangle.” Below linea arcuata, the preparation is continued in the preperitoneal space, if necessary, extended to the spatium Retzii behind the pubic bone. In the case of defects close to the xyphoid, the preparation is extended into the retroxyphoidal space by dissection of the posterior rectus sheath from the xyphoid [20, 21]. The repair started with closure of the posterior rectus sheath with an absorbable continuous suture (Vicryl 1-0, Ethicon Inc., Somerville, NJ, USA). The size of the defect was documented with width and length. According to randomization, the allocated mesh was cut to a size that ensured an overall overlap of a minimum of 5 cm in all directions. Fixation of the mesh was performed with interrupted stitches with a nonabsorbable suture (Prolene 2-0, Ethicon Inc., Somerville, NJ, USA) 1 cm from the mesh edge with no more than 2 cm between each stitch. One or two drainages were placed onto the mesh before closure of the anterior fascia of the rectus sheath with a running, nonabsorbable loop suture (Prolene 1-0, Ethicon Inc., Somerville, NJ, USA). Fascia closure was performed with a suture length to wound length ratio of 4:1. Drainage and skin closure were performed according to each center’s standard procedure.

### Statistics and randomization

#### Sample size

The sample size calculation was based on an expected difference with respect to the time until the first occurrence of foreign body sensation within the first year after surgery. It was expected that within the first year after surgery, foreign body sensation occurs in 40% of the cases with Premilene^®^ mesh and in 20% of the cases with Ultrapro^®^ mesh [22]. With these assumptions, a total sample size of 90 patients in each treatment group was calculated (significance level, 5%; power, 80%; accrual time, 1 year; follow-up time, 2 years; dropout rate, 20%; computation with nQuery advisor, version 4.0, ^©^Statistical Solutions).

#### Randomization, allocation concealment, and blinding

The randomization was performed by letters included in sealed envelopes with consecutive patient numbers indicating the mesh to be applied. The randomization codes were balanced in permuted blocks of varying size, stratified by surgeon. Randomization lists were generated by the documentation center using S-PLUSTM (Version 6.1 Windows Professional Release 1^©^, Mathsoft Inc.). Randomization by envelope was preferred because in a relevant proportion, inclusion of the patients in the study could be confirmed only during surgery. Although the date and time of opening of the randomization envelope had to be documented on the randomization letter, bias due to allocation concealment could not be excluded. Accordance to the guidelines in handling the envelopes was verified at monitoring visits. In addition, the randomization list was kept sealed in the documentation center. Intraoperative blinding of the surgical team could not be implemented for practical reasons. Patients, investigators, and assessors were blinded during the follow-up process. Thus, formally, the study has to be considered an open-label study.

#### Statistical methods

Meshes were compared based on the time until the first occurrence of foreign body sensation within the first year after surgery using a Cox proportional hazards model (factor “mesh”; block factor “surgeon”; no interaction; test of the main effect “mesh”; two-sided 5% significance level). The secondary endpoints “rate of hematoma after 5 days” and “rate of chronic pain after 24 months” were analyzed using the Mantel–Haenszel chi-square test stratified by surgeon. In addition, the PCS and MCS summary scores of the SF-36^®^ life quality questionnaire computed after 4 months were examined by 2-factor analysis of covariance (factor “mesh”; block factor “surgeon”; no interaction; baseline value as covariate; test of the main effect “mesh”). Two-factor analysis of covariance (factor “mesh”; block factor “surgeon”; interaction mesh*surgeon; covariate “VAS baseline”; test of the main effect “mesh”) was also used for statistical evaluation of VAS scores observed after 24 months within the subgroup of patients with chronic pain after 24 months. The secondary endpoint criteria “rate of hematoma requiring surgery after 5 days,” “rate of wound infection after 24 months,” and “rate of seroma after 24 months” were analyzed in a descriptive manner only. Surgeons who contributed fewer than 5 patients were combined. We computed two-sided *p* values only for comparison with the two-sided 5% significance level. Statistical analysis was performed using SAS (The SAS System; Release 9.1.3 SP 4; SAS Institute Inc., Cary, NC, USA) on Windows XP SP 3 (Microsoft Corp., Redmond, WA, USA). SAS PROC FREQ, PROC GLM, and PROC TPHREG were used to perform statistical tests. Database access was performed using an SQL database (PostgreSQL) via the ODBC interface.

## Results

### Baseline data

Between September 2004 and June 2006, 19 surgeons in 8 centers recruited 181 patients who met the criteria for statistical evaluation of the primary endpoint. The median number of patients per center was 20.5 (range 5–47) and per surgeon was 9 (range 1 to 30). The mesh allocation was balanced. The difference in the number of patients per mesh was 2 for one of the 19 surgeons and at most 1 for the remaining 18 surgeons. The baseline data regarding patient characteristics within the treatment groups are listed in Table 2. Baseline characteristics were balanced between the two meshes. However, the proportion of smokers was 6.9% higher in the Premilene^®^ group than in the Ultrapro^®^ group (Ultrapro^®^: 20.9%, Premilene^®^: 27.8%).

**Table 2:** Patient baseline characteristics

Characteristic	Ultrapro^®^ (*n*=91)	Premilene^®^ (*n*=90)
Male [*n* (%)]	52 (57.1)	48 (53.3)
Age (years) [mean (SD)]	62.2 (12.1)	62.7 (11.7)
Body mass index [mean (SD)]	29.7 (5.0)	29.4 (4.9)
Primary incisional hernia [*n* (%)]	78 (85.7)	80 (88.9)
Smokers [*n* (%)]	19 (20.9)	25 (27.8)
Obesity [*n* (%)]	41 (45.1)	43 (47.8)
COPD [*n* (%)]	12 (13.2)	10 (11.1)
Cortisol therapy [*n* (%)]	3 (3.3)	1 (1.1)
Diabetes [*n* (%)]	14 (15.4)	11 (12.2)
Malnutrition [*n* (%)]	1 (1.1)	1 (1.1)
Renal failure [*n* (%)]	5 (5.5)	5 (5.6)
Medication [*n* (%)]	73 (80.2)	73 (81.1
Operation time (min) [median (range)]	104 (42–257)	105 (35–270)

*n*, no. of observations; *SD*, standard deviation

### Participant flow

**Fig. 1:**
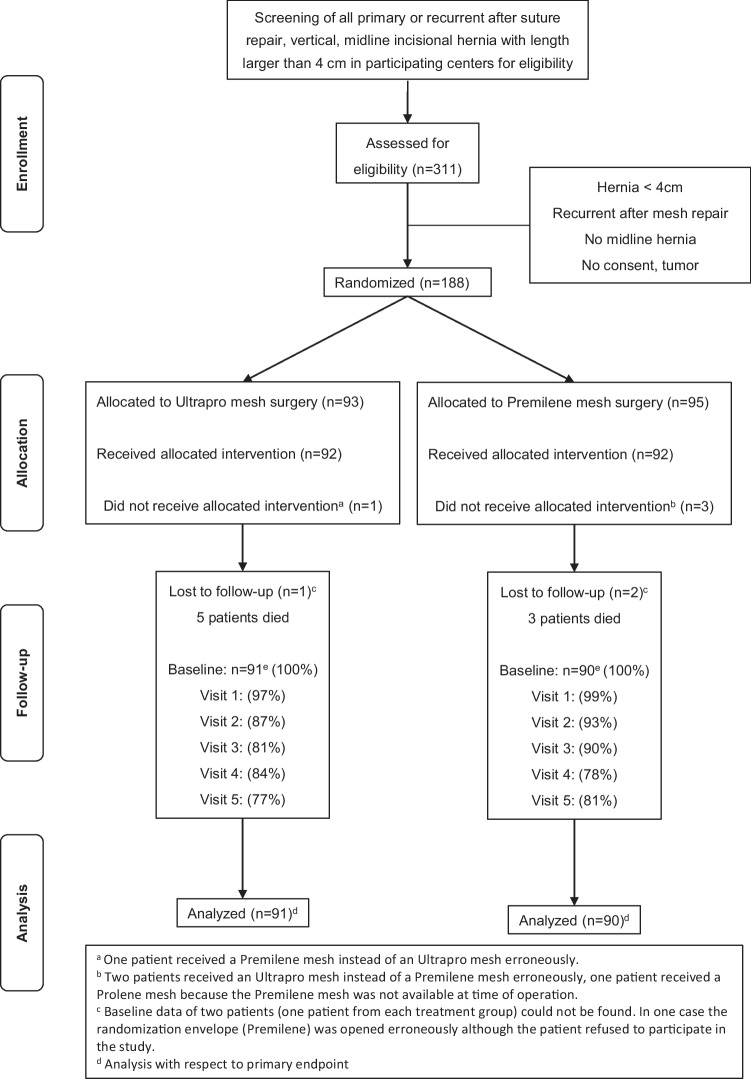
Flow diagram

The patient flow is displayed in Fig. 1. In total, 311 patients were assessed for eligibility. A total of 184 patients were included in the study. One patient had to be excluded prior to randomization because the randomization envelope was lost. In other cases, the randomization process was not correctly conducted. One patient received a Premilene^®^ mesh instead of an Ultrapro^®^ mesh. Two patients received Ultrapro^®^ mesh instead of Premilene^®^ mesh, and one patient received Prolene^®^ mesh because the Premilene^®^ mesh was not available at the time of operation. Out of the 184 cases, one additional patient in the Premilene^®^ group had to be excluded from statistical analysis because the randomization envelope was opened, although the patient refused to participate in the study prior to randomization. For one patient in each intervention groups, no postoperative documentation was available. Thus, according to the intention-to-treat (ITT) principle, the data of 181 (98.4%) patients (Ultrapro^®^, 91 patients; Premilene^®^, 90 patients) met the criteria for the statistical evaluation of the primary endpoint. Of these, 5 patients (5.5%) died in the Ultrapro^®^ group, and 3 patients (3.3%) died in the Premilene^®^ group within the follow-up, independent of the incisional hernia repair performed. For one patient, the follow-up visit after 24 months deviated by approximately 100 days from the prespecified time frame. In all other cases, the deviation was not as extreme as for this patient (for 21-day visits, at most ±2 days; for 4-month visits, at most ±2 days; for 24-month visits, at most ±1 day).

### Surgical details

All procedures were performed under general anesthesia. The mean operating time was 104 min (range 42–257 min) in the Ultrapro^®^ group and 105 min (range 35–270 min) in the Premilene^®^ group, with no significant difference between the two groups. There was also no significant difference among the surgeons regarding the operating time. The mean time spent in the hospital was 10 days for Ultrapro^®^ and 10 days for Premilene^®^.

### Outcomes and estimation

#### Primary endpoint: foreign body sensation

The mean follow-up period was similar between the two mesh groups (Ultrapro^®^, 648.5 (± 241.1) days; Premilene^®^, 657.1 (± 235.3) days). The results obtained for the primary and secondary endpoints are given in Table 3. The data of 91 cases in the Ultrapro^®^ group and of 90 cases in the Premilene^®^ group could be used for the analysis of the primary endpoint criterion. We observed foreign body sensation within the first year after surgery, without statistically significant differences, in 25 cases (27.5%) in the Ultrapro^®^ group and in 29 cases (32.2%) in the Premilene^®^ group (hazard ratio “Ultrapro vs. Premilene,” −4.7%; 95% CI (−18.1; 8.6); *p* = 0.9172). The time until first occurrence of foreign body sensation within the first year did not differ significantly between the Ultrapro^®^ and Premilene^®^ mesh groups (hazard ratio “Premilene vs. Ultrapro,” 1.03; 95% confidence interval [CI] (0.60; 1.77); *p* = 0.9127). Moreover, there was no statistically significant mesh-by-surgeon interaction effect on the primary endpoint (*p* = 0.8994).

**Table 3:** Analysis of primary and secondary endpoints

Characteristic	Time of analysis	Ultrapro (*n*=91)^#^		Premilene (*n*=90)^#§^		Treatment difference (95% CI)	*p value*
		Baseline	Final	Baseline	Final		
Foreign body sensation^a^ [*n* (%)]	12 months	—	25 (27.5)	—	29 (32.2)	−4.7 (−18.1; 8.6)	0.9127^f^
Hematoma [*n* (%)]	5 days	—	19 (20.9)	—	21 (23.3)	−2.5% (−14.5; 9.6)	0.7295^g^
Hematoma requiring surgery [*n* (%)]	5 days	—	0 (0.0)	—	1 (1.1)	—	*—*
SF-36 life quality scores [mean (SD)]							
PCS^b^	4 months	42.7 (11.5) [*n*=83]	44.8 (9.9) [*n*=80]	41.6 (10.8) [*n*=80]	43.7 (9.9) [*n*=79]	1.1 (−2.0; 4.2)	0.4895^h^
MCS^c^	4 months	49.0 (12.4) [*n*=83]	50.3 (11.8) [*n*=80]	47.2 (11.8) [*n*=80]	49.4 (11.9) [*n*=79]	0.9 (−2.9; 4.6)	0.9377^h^
Chronic pain^d^ [*n* (%)]	24 months	—	18 (19.8)	—	16 (17.8)	2.0 (−9.4; 13.4)	0.7202^g^
VAS score^e^ [mean (SD)]	24 months	3.02 (3.00) [*n*=18]	3.62 (2.24) [*n*=18]	4.48 (3.39) [*n*=16]	3.14 (1.50) [*n*=16]	0.48 (−0.87; 1.83)	0.1152^i^
Wound infection [*n* (%)]	24 months	—	1 (1.1)	—	0 (0.0)	—	*—*
Seroma [*n* (%)]	24 months	—	2 (2.2)	—	1 (1.1)	—	*—*
Recurrence	24 months	—	5 (5.5)	—	4 (4.4)		

*n*, no. of observations; *SD*, standard deviation; ^#^one patient excluded from each intervention group due to missing postoperative documentation; ^§^one patient of the Premilene group excluded from analysis due to erroneous opening of the randomization envelope although the patient refused to participate in the study

^a^Within the first 12 months postoperative

^b^Physical component summary of SF-36 life quality scores; missing values at visit “4 months” were substituted by LOCF method

^c^Mental component summary of SF-36 life quality scores; missing values at visit “4 months” were substituted by LOCF method

^d^Defined as pain persisting over a period of at least 3 months; missing values at final visit “24 months” were substituted by according values obtained at visit “12 months” (if possible)

^e^Analyzed for subgroup of 34 patients (Ultrapro, 18 patients; Premilene, 16 patients) with chronic pain at final visit “24 months”; missing values at final visit “24 months” were substituted by according values obtained at visit “12 months” (if possible)

^f^Cox regression model (factor “mesh”; block factor “surgeon”; no interaction)

^g^Mantel–Haenszel chi-square test stratified by surgeon

^h^Analysis of covariance model (factor “mesh”; block factor “surgeon”; no interaction; baseline value as covariable)

^i^Analysis of covariance model (factor “mesh”; block factor “surgeon”; interaction mesh*surgeon; baseline value as covariable)

#### Wound complications

After 24 months, seroma occurred in two patients (2.2%) in the Ultrapro^®^ group and in one patient (1.1%) in the Premilene^®^ group. The postoperative 5-day hematoma rates were 19/91 (20.9%) in the Ultrapro^®^ group and 21/90 (23.3%) in the Premilene^®^ group. This difference in hematoma rates was not statistically significant (rate difference “Ultrapro vs. Premilene,” −2.5%; 95% CI (−14.5; 9.6)). The stratified odds ratio (OR) between Premilene^®^ mesh and Ultrapro^®^ mesh was 1.13 (95% CI (0.55; 2.34)). The Mantel–Haenszel chi-square test stratified by surgeon yielded a statistically nonsignificant test result (*p* = 0.7295). The Breslow–Day test for homogeneity of the ORs across surgeons did not indicate a statistically significant difference (*p* = 0.1070). In the Premilene^®^ group, reoperation because of hematoma was performed in one patient, while in the Ultrapro^®^ group, no surgery due to hematoma appearance was necessary.

#### Quality of life (SF-36)

Owing to missing values, PCS and MCS baseline measurements were available for only 83 (91.2%) patients in the UItrapro^®^ group and for only 80 (88.9%) patients in the Premilene^®^ group. The LOCF method was applied for substitution of missing PCS and MCS values at the 4-month visit. With these replacement data, a total of 80 patients (87.9%) in the Ultrapro^®^ group and 79 patients (87.8%) in the Premilene^®^ group could be used for analysis of the secondary endpoint criteria PCS and MCS. Mean PCS values after 4 months were slightly higher in the Ultrapro^®^ group (44.8; ± 9.9) than in the Premilene^®^ group (43.7; ± 9.9). This difference in mean PCS values between the two meshes was not statistically significant (mean difference, 1.1; 95% CI (−2.0; 4.2), *p* = 0.4895). Based on the protocol, we fitted a 2-factor analysis of covariance (ANCOVA) model to the PCS measurements observed after 4 months, using “surgeon” as a stratification factor. Moreover, we included PCS baseline measurements as a covariable in the ANCOVA model. The corresponding *F* test for the main effect of “mesh” demonstrated a statistically nonsignificant difference in 4-month PCS measurements between the Ultrapro^®^ and the Premilene^®^ mesh (*p* = 0.4895). The mesh-by-surgeon interaction gave no signs of a dependence for PCS differences between meshes across surgeons (*p* = 0.2025). PCS baseline values appeared to have a statistically significant impact on corresponding values observed after 4 months (*p* < 0.0001), while no statistically significant effect of “surgeon” was shown (*p* = 0.3435). Analysis of MCS measurements yielded similar results. Similar to the previous results, the mean MCS values after 4 months were slightly elevated in the Ultrapro^®^ group (50.3; ±11.8) compared to the Premilene^®^ group (49.4; ±11.9). Thus, the mean change in MCS values between the two meshes was 0.9 (95% CI (−2.9; 4.6)) and therefore not statistically significant. Applying the same ANCOVA model as before to the MCS data, no significant difference in 4-month MCS measurements between the Ultrapro^®^ mesh and the Premilene^®^ mesh was found (*p* = 0.9377). Moreover, neither a statistically significant surgeon main effect (*p* = 0.6230) nor a statistically significant mesh-by-surgeon interaction effect (*p* = 0.3968) on 4-month MCS measurements was detected, while those values again depended statistically significantly on the corresponding baseline measurements (*p* < 0.0001). The mesh-by-surgeon interaction gave no signs of a dependence for PCS differences between meshes across surgeons (*p* = 0.2025). MCS baseline values appeared to have a statistically significant impact on corresponding values observed after 4 months (*p* < 0.0001), while no statistically significant effect of “surgeon” was shown (*p* = 0.3435).

#### Chronic pain

Chronic pain at the final visit (24 months) was diagnosed in 18 patients (19.8%) in the Ultrapro^®^ group and in 16 patients (17.8%) in the Premilene^®^ group. This difference of 2% (95% CI (−9.4; 13.4)) in the occurrence rates of chronic pain between the two meshes was negligible. The stratified OR between the Premilene^®^ mesh and the Ultrapro^®^ mesh was 0.88 (95% CI: 0.42; 1.83). The Mantel–Haenszel chi-square test stratified by surgeon yielded a statistically nonsignificant test result (*p* = 0.7202). The Breslow–Day test for homogeneity of the ORs across surgeons indicated a statistically significant difference (*p* = 0.0198). Further investigation of this result demonstrated that the rate of chronic pain after 24 months was increased in the Premilene^®^ group for 7 surgeons, contributing a total of 86/181 patients (47.5%), and elevated in the Ultrapro^®^ group for another 4 surgeons, contributing 66/181 (36.5%) patients. None of the patients recruited by the remaining surgeons exhibited chronic pain after 24 months. Furthermore, within the subgroup of 34 patients diagnosed with chronic pain after 24 months, we analyzed VAS (visual analog scale) scores. According to the statistical evaluation of the secondary endpoint criteria PCS and MCS, we used the LOCF method to replace missing VAS values at 24 months with their corresponding measurements observed after 12 months. Therefore, data from all 34 patients in the chronic pain subgroup were used for comparison of VAS measurements obtained after 24 months between the two meshes. Interestingly, the mean baseline VAS was clearly increased in the Premilene^®^ group (4.48; SD, 3.39) compared to the Ultrapro^®^ group (3.02; SD, 3.00), while the mean VAS values obtained after 24 months were higher in the group treated with Ultrapro^®^ mesh (3.62; SD, 2.24) than in patients treated with Premilene^®^ mesh (3.14; SD, 1.50). Nevertheless, this obvious change in mean VAS between the two meshes failed to be statistically significant (mean difference, 0.48; 95% CI (−0.87; 1.83)). We investigated the effect of mesh type on VAS by fitting a 2-factor analysis of covariance (ANCOVA) model to the VAS measurements observed after 24 months, using “surgeon” as a stratification factor. Moreover, we included VAS baseline measurements as a covariable in the ANCOVA model. The corresponding *F* test for the main effect of “mesh” demonstrated a statistically nonsignificant difference in VAS measurements after 24 months between the Ultrapro^®^ and the Premilene^®^ mesh (*p* = 0.1152). VAS baseline values did not show a statistically significant impact on VAS values after 24 months (*p* = 0.1945), but there was a statistically significant main effect of “surgeon” (*p* = 0.0375) and a statistically significant mesh-by-surgeon interaction effect (*p* = 0.0037). Note that the analysis of VAS is conditioned on the occurrence of chronic pain. Thus, the dataset used for this analysis reflected only 18.8% (34/181 patients). Because all surgeons contributed fewer than 5 patients in this dataset, the degrees of freedom to estimate the treatment-by-surgeon interaction effect were low (i.e., *d* = 5). Therefore, the test for the treatment-by-surgeon interaction effect lacked power.

#### Recurrence

We observed a new fascial defect during the follow-up investigation in 11 patients; 9 defects were at the site of mesh repair and 2 were new concomitant hernias distant from mesh repair. Recurrences occurred in 5 (5.5%) patients with Ultrapro^®^ repair and in 4 (4.4%) patients with Premilene^®^ repair. The location of the recurrence was at the cranial edge of the mesh in 5 patients (3× Ultrapro^®^, 2× Premilene^®^). During the follow-up ultrasound investigation, we found concomitant rectus diastasis with a width of more than 2.5 cm in 4 of the 5 recurrences. The other cranial recurrences showed insufficient mesh subduction behind the linea alba. The remaining 4 recurrences occurred through the mesh. In one patient, emergency relaparotomy through the mesh became necessary because of ileus 5 months after the initial mesh repair. Closure of the relaparotomy was performed with a resorbable suture leading to hernia recurrence within 6 weeks. Two other patients suffered from a wound infection, one leading to necrotizing fasciitis and one to a wound sinus with abscess formation, making partial mesh resection necessary. One patient suffered a central hernia recurrence by mesh rupture. In this case, the mesh was incorrectly placed. It was rotated by 90°, resulting in a disrupted craniocaudal orientation of the mesh.

## Discussion

Mesh-reinforced hernia repair is considered the gold standard in incisional hernia repair [23], but the benefits attributed to mesh implantation might be partly offset by mesh-related side effects such as foreign body sensation or chronic pain [24]. Therefore, different mesh types have been developed to improve biocompatibility and decrease mesh-related complications. Experimental data indicate that small-pore meshes lead to a pronounced foreign body reaction compared to large-pore meshes [25–27]. This is mostly due to increased inflammation and fibrosis following the local cellular and tissue reaction to fibers within meshes [16, 28]. Eventually, the entire mesh is entrapped in scar tissues, leading to less elasticity and probably to a more pronounced foreign body sensation [29–31]. In addition to using larger pores, composite meshes with partially resorbable materials were introduced to further improve biocompatibility [32–34]. However, in this RCT, we did not identify any significant difference in surgical or patient-reported outcomes between a small-pore and a partially absorbable larger-pore composite mesh. Clinical studies describing differences between small- and large-pore meshes in incisional hernia repair are scarce. In a prospective observational cohort study comparing small-pore *versus* large-pore meshes, foreign body sensation was lower in large-pore meshes (38% vs. 21%), but this difference was not statistically significant 3 years after surgery [35]. Similar to foreign body sensation, the evaluation of chronic pain 24 months after surgery did not differ between groups. Further investigation of the patients with chronic pain resulted in a statistically significant main effect of “surgeon” and a statistically significant mesh-by-surgeon interaction effect. Even though this analysis only reflects 18.8% of all patients, the data support the assumption that technical aspects and a surgeon’s skills are more important than the mesh that was implanted. However, the importance of surgical expertise was also emphasized in a register study by Pereira et al. They concluded that surgery for incisional hernia was associated with better results when conducted in a specialized abdominal wall unit [36].

As this study was designed based on the experiences of the “Vypro” trial addressing the disappointing results with an unexpectedly high recurrence rate of 12% [10], a closer look at the surgical outcome is also necessary. One possible explanation for the high recurrence rate in the “Vypro” trial was certain impairments in the study protocol, leaving too many factors of the procedure to each clinic’s standard procedure. Therefore, the technical aspect of achieving sufficient mesh subduction behind the linea alba, the preparation of the “fatty triangle,” was developed and published [20, 21] and integrated into the new study protocol as one of the crucial steps of the procedure. The overall recurrence rate in this study was 5% after 24 months (Ultrapro^®^, 5.5% and Premilene^®^, 4.4%). In comparison to the Vypro trial and the Luijendijk study [23, 37] or registry data, which reported recurrence rates between 12 and 30% [38, 39], the standardization of the procedure seems to reduce hernia recurrence. Taking a closer look at the 9 patients with recurrent hernia, two recurrences were related to technical problems. In one case, the mesh was positioned incorrectly (rotated by 90°), and in the second case, the cranial overlap was insufficient. In another patient who needed emergency relaparotomy, the fascia was closed with a resorbable suture prior to hernia recurrence. Hence, 30% of the recurrences could be explained by technical problems or mistakes, underlining the importance of a high surgical standard. Two further recurrences were related to a complicated postoperative course with partial mesh explantation. The remaining recurrences in our study were associated with rectus diastasis. In these cases, the recurrences developed at the border of the mesh, indicating an insufficient overlap underneath healthy tissue. The impact and correlation of hernia recurrence and rectus diastasis have not yet been resolved. A recent retrospective analysis of patients with umbilical and epigastric hernias identified a significant increase in hernia recurrence in patients with rectus diastasis [40]. High-quality RCTs with a focus on rectus diastasis and hernia recurrence are missing, and we will need further investigation to finally answer this question. Nevertheless, rectus diastasis should always be considered a risk factor in incisional hernia repair. Currently, we extend our mesh augmentation in these patients far beyond the initial facia scar all the way behind the xiphoid. Unfortunately, the issue of rectus diastasis in incisional hernia repair was not addressed in the study protocol, and an extension into the retroxiphoidal space was only recommended if necessary.

Even though the surgical outcome was improved in our study compared to the literature, patient-reported outcomes such as foreign body sensation or chronic pain are becoming increasingly important in evaluating surgical success. The incidences of surgery-specific and patient-centered outcomes reported in the literature vary dramatically. A recently published systematic review of outcome reporting in incisional hernia surgery demonstrated significant heterogeneity in defining postoperative complications and outcomes [41]. However, in our study, we observed foreign body sensation in 27.5% in the Ultrapro^®^ and 32.2% in the Premilene^®^ mesh group and chronic pain in 19.8% (Ultrapro^®^) and 17.8% (Premilene^®^). In a recently published study, patient-reported outcomes after incisional hernia repair using the Carolina Comfort Scale (CCS) mesh sensation were observed in 23% of the included patients. According to the same study, 44% of patients reported pain, and 53% were symptomatic [42]. Even though this study included open and laparoscopic approaches, it shows that patient-reported outcomes such as foreign body sensation or chronic pain still hamper quality of life after incisional hernia repair, and rates for foreign body sensation and chronic pain are comparable to our results. Other data report similar rates of foreign body sensation (21–38%) or pain in the operation area [35, 43]. These older data show that we have experienced progress in improving surgery-specific outcomes such as recurrence rates, but patient-reported outcomes need to be addressed in future research activities. Today, in addition to improved mesh material, new minimally invasive surgical procedures, such as the endoscopically assisted mini- or less-open sublay (MILOS) concept or the extended totally extraperitoneal approach (eTEP), can reduce surgical trauma and might improve patient-reported outcomes [44, 45].

Some limitations of this RCT must be considered. First, the study was conducted between 2004 and 2008 and the publication of the study was delayed. Changes in the staff and the responsibilities of the surgical department of our university were the main reasons. Nevertheless, we are convinced that the presented data are still of relevance for the surgical community, but the results must be considered carefully. Documentation, surgical classifications, and perioperative treatment strategies have developed since finishing the study. Today, we prefer a validated hernia-specific questionnaire such as the CCS [46, 47] to describe patient-reported outcomes. Furthermore, we would use the EHS classification for incisional abdominal wall hernias to report hernia characteristics [48] and the Clavien–Dindo classification for complications [49]. Finally, the results of the secondary endpoints, such as recurrences, must be interpreted carefully because the study was powered for foreign body sensation. Furthermore, we would include recommendations for patients with rectus diastasis in the study protocol for future trials.

## Conclusions

In this multicenter randomized controlled trial, we did not find any differences between the use of a small-pore polypropylene mesh (Premilene^®^) and a larger-pore composite mesh (Ultrapro^®^) in a standardized open retromuscular meshplasty for incisional hernia repair regarding patient-reported outcomes and surgery-specific outcomes. Even though a relevant proportion of the included patients reported foreign body sensations, our trial was not able to show the superiority of the larger-pore composite mesh. It seems that a high standardization of the surgical procedure improves the surgical outcome, such as recurrence rates, but patient-reported outcomes, such as foreign body sensation or chronic pain, remain relevant issues in hernia repair independent of the mesh. Future studies should employ a high standardized outcome set, including surgical and patient-related parameters, in addition to a high standardization of the surgical procedure.

## Data Availability

The full trial protocol and data used to support the findings of this study are available from the corresponding author and the principal investigator upon request.
